# Myopia Management in Hong Kong

**DOI:** 10.3390/jcm14030698

**Published:** 2025-01-22

**Authors:** Han-Yu Zhang, Fang-Yu Xu, Kenneth Ka King Liu, Yan-Pui Chan, Amy Chow, Deborah Jones, Carly Siu Yin Lam

**Affiliations:** 1 Centre for Eye and Vision Research (CEVR), 17W Hong Kong Science Park, Hong Kong SAR, China; hanyuzhang@nankai.edu.cn (H.-Y.Z.); queenie.xu@polyu.edu.hk (F.-Y.X.); yan-pui.chan@polyu.edu.hk (Y.-P.C.); amy.chow@uwaterloo.ca (A.C.); debbie.jones@uwaterloo.ca (D.J.); 2 School of Medicine, Nankai University, Tianjin 300071, China; 3 Centre for Myopia Research, School of Optometry, The Hong Kong Polytechnic University, Hong Kong SAR, China; kennethkk.liu@polyu.edu.hk; 4 School of Optometry & Vision Science, University of Waterloo, Waterloo, ON N2L 3G1, Canada

**Keywords:** myopia management, pre-myopia, myopia, myopia control, retrospective review

## Abstract

**Objectives:** We aimed to investigate how optometrists in Hong Kong are managing myopic and “pre-myopic” children. **Methods:** Clinical files for children aged 6 to 10 years old who had eye examinations from 2017 to 2021 were retrospectively reviewed. Children were grouped by the initial spherical equivalent refractive error (SER) as myopes or pre-myopes. The demographic data, refractive error, and myopia management recommended by the optometrists were analyzed. **Results:** A total of 1,318 children (859 myopes and 459 pre-myopes) from ten clinics in Hong Kong were included. Over 5 years, myopia management recommendations shifted significantly (*p* < 0.001). In 2017, only 18.4% of children were recommended to pursue myopia control (MC), increasing to 42.8% by 2021. The use of MC spectacle lenses increased from 7.3% in 2017 to 36.8% in 2021, becoming the most recommended option. Orthokeratology, MC contact lenses, and atropine remained stable at less than 5% over this period. Children recommended for MC approaches had significantly more myopia than those recommended single-vision lenses or monitoring (*p* < 0.05). Age of the first visit significantly correlated with SER change from the first visit to the next recommendation update for pre-myopes (r = 0.27, *p* = 0.013) but not for myopes. **Conclusions:** From 2017 to 2021, myopia management patterns in Hong Kong shifted significantly, with more children being recommended for myopia control. MC spectacle lenses emerged as the most commonly recommended method. Younger pre-myopes at their first visit were more likely to have earlier management updates.

## 1. Introduction

The prevalence of myopia has increased over the past 50 years, especially in Asia [1,2,3]. It has been estimated that 49.8% and 9.8% of the world’s population will suffer from myopia and high myopia, respectively, by 2050 [3]. However, with excessive axial elongation and a bigger eye size, high myopia is associated with retinal detachment, myopic macular degeneration, cataracts and glaucoma, which impact the quality of life and increase risks of visual impairment [4,5]. Myopia is a global concern, with an increased burden in both economic and public health [6,7]. Normal physiological myopia develops into high myopia or even pathological myopia if there is no appropriate control intervention [8]. It is important to formulate public health policies and study interventions to prevent myopia onset and to retard myopia progression.

Both optometrists and ophthalmologists provide primary eye care and play an important role in myopia management. In 2019, it was documented that the practice patterns of pediatric ophthalmologists and treatment efficacy varied significantly across the world [9]. Leshno and colleagues found that almost all ophthalmologists used at least one form of effective treatment to treat myopia. European physicians offered the lowest rate of myopia treatment compared with other global regions (85% vs. mean 97%) [9]. Rates of effective optical treatment varied significantly between locations, from 16% in Central–South America to 56% in the Far East [9]. Moreover, the concern of eye care and engagement in myopia control has increased in recent decades. However, the majority of myopia management methods are still using single-vision spectacle lenses [10,11,12].

Hong Kong has one of the highest myopia prevalences in the world [1,13,14], but little is known about how optometrists manage myopia for children in Hong Kong, despite optometrists being one of the primary providers of primary eye care in Hong Kong. Through this investigation, we seek to provide an insight into current clinical practices and trends in myopia management, highlighting how early interventions and treatment modifications are being applied to address the growing prevalence of myopia in Hong Kong’s youth. Therefore, this study aims to investigate how optometrists in Hong Kong are managing their young myopic and “pre-myopic” patients.

## 2. Methods

This was a retrospective chart review of records from 10 practices from Hong Kong Island, Kowloon and New Territories in a variety of settings. Patient charts were reviewed at each location by a site investigator or designated research personnel. This study was approved by the Human Subjects Ethics Sub-Committee (HSESC) of Hong Kong Polytechnic University. Participating optometrists were provided with the background of the research, and informed consent statements were obtained from each participant. The study was designed to be in conformance with the ethical principles in the Declaration of Helsinki and with the ICH guidelines for Good Clinical Practice (GCP). To recruit the clinical practices, we sent an invitation letter to the Hong Kong Association of Private Practice Optometrists (HKAPPO), the Hong Kong Optometric Association (HKOA), and the Hong Kong Society of Professional Optometrists (HKSPO).

The protocol was designed for the simultaneous collection of data in Hong Kong and Canada. Charts were selected based on age and spherical equivalent refractive error (SER) at their initial visit. Children were then grouped as myopes (SER ≤ −0.50D) or pre-myopes (−0.50D < SER ≤ +0.75D). Five unique files were selected for each age with a presenting appointment in one of the calendar years selected (see Supplementary Figure S1). More than 5 files may have been reviewed to satisfy the inclusion criteria.

Patient charts were included if the following applied:

1. The patient was aged 6–10 inclusively.
2. The patient had at least one appointment in any year from 2017 to 2021 inclusively.
3. The patient saw the same primary clinician for the majority of their appointments.
4. One group is pre-myopes, and they have a refractive error ≤ +0.75D in each principal meridian; the other group is myopes and they have a refractive error of ≤−0.50D in each principal meridian.
5. The patient had no significant binocular vision issues.
6. The patient had correctable acuity to at least 20/30 in each eye.
7. The patient had no development impairment or special needs.
8. The patient had no ocular or systemic disease.

Spherocylindrical refraction measurements regarding spherical power (S), cylindrical power (C), and axis (θ) were converted into a power vector by a conventional formula for analysis [15].

SER = S + C/2

J_0_ = − (C/2) cos(2θ)

J_45_ = − (C/2) sin(2θ)

## 3. Data Analysis

Statistical analysis was performed by using SPSS (version 26.0, IBM, Armonk, NY, USA). Data normality was tested by Shapiro–Wilk tests, and non-parametric methods were used for data not normally distributed. Normally distributed data were presented as mean ± SD; otherwise it was presented as median values (minimum to maximum).

The Mann–Whitney test was performed to compare the difference between myopic and pre-myopic groups. Pearson’s chi-squared test was used to compare the sex distribution of myopes and pre-myopes. The crosstabs chi-squared test with the Bonferroni correction was applied to examine the relationship between myopia management and visiting years. The refractive error and age among different myopia management strategies were analyzed by the Kruskal–Wallis test. The association between the time for the first modification of myopia management and age and refractive error was analyzed using the Pearson correlation. *p* < 0.05 was set as the significance level.

## 4. Results

### 4.1. Demographic Characteristic Data

A total of 10 clinics, with 7 clinics belonging to different private entities and 3 clinics belonging to the Optometry Centre of the university, were included. Twenty-three optometrists (none of them worked at more than one practice) participated in the survey and provided the medical records. Their graduation year ranged from 1983 to 2016, which was normally distributed (*p* = 0.20, see Supplementary Figure S2).

There were 1318 eligible children (859 myopes and 459 pre-myopes), collected from 10 clinics. Myopes had significantly more positive J_0_ (*p* < 0.001) and negative J_45_ (*p* = 0.037) than pre-myopes. Myopes had significantly more myopic refractive errors, longer axial lengths (ALs) and older ages (*p* < 0.001) than pre-myopes. No significant difference was evident for best-corrected visual acuity (BCVA) or sex distribution between myopes and pre-myopes (Table 1).

### 4.2. Recommended Myopia Management

With respect to the recommended myopia management approach, nine methods were summarized: monitor without correction (MWC), no optical or pharmacological devices, single-vision (SV) spectacle lenses, bifocal/progressive additional lenses (PALs), myopia control (MC) spectacle lenses, myopia control contact lenses (MCCLs), orthokeratology (ortho-K) lenses, atropine, or unclassified (Table 2).

**Figure 1:**
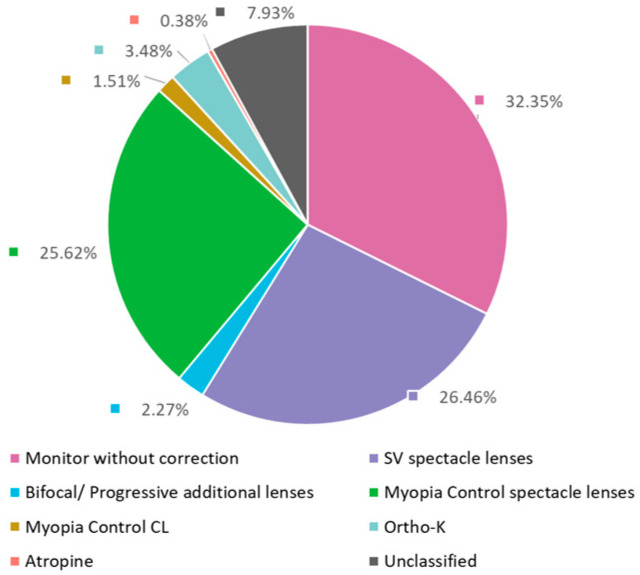
Recommended myopia management for children at the initial visit from 2017 to 2021.

At the first visit, the top three recommended myopia management from optometrists were MWC (32.35%), prescribed SV spectacle lenses (26.46%), and myopia control spectacle lenses (25.62%). And 33.26% of children were recommended to conduct myopia control strategies (including myopia control spectacle lenses, PALs, MCCL, Ortho-K lenses, and atropine) (Figure 1).

**Figure 2:**
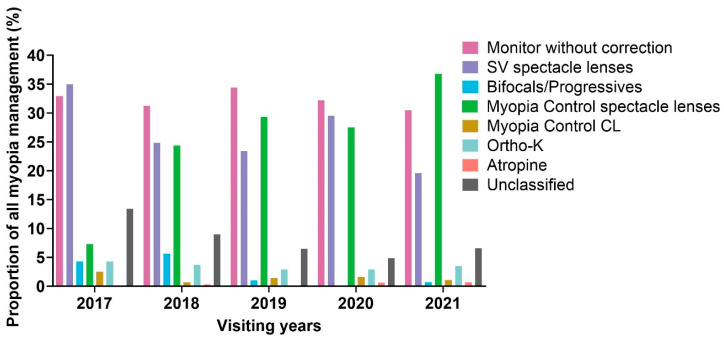
Changes in recommended myopia management from 2017 to 2021.

From 2017 to 2021, the recommended myopia management patterns changed significantly (*p* < 0.001, Figure 2, see Supplementary Figure S3). Only 18.4% of children were recommended to take myopia control in 2017, while the proportion increased to 42.8% in 2021. The recommendation of MC spectacle lenses accounted for 7.3% in 2017 and increased to the highest proportion with 36.8% in 2021. The recommendation of Ortho-K lenses and MCCL was stable at less than 5% approximately over 5 years. Atropine was the least recommended method, which was less than 1%. Children recommended myopia control approaches had significantly more myopia than children recommended SV lenses and those offered no recommendation (*p* < 0.05).

**Figure 3:**
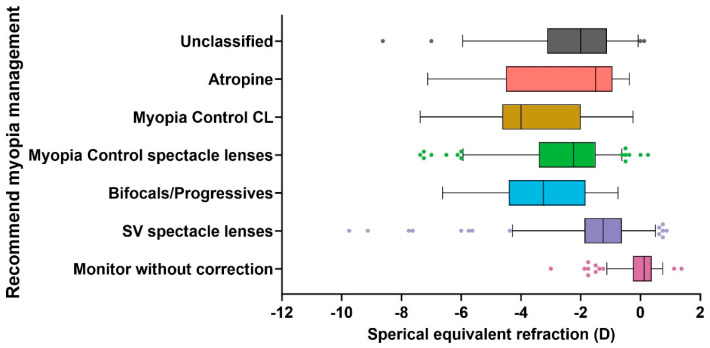
SER distribution among recommended myopia management of all children. Box–whisker plot with 2.5% to 97.5% percentile. The scattered points are referred to as Outliers.

Significant differences in SER distribution among the recommended management strategies were observed (Kruskal–Wallis, *p* < 0.001, Figure 3). The refractive error in MWC showed the fewest myopia cases among all management plans (*p* < 0.001), with a median of 0.125D (−3D, +1.38D). MCCL with a median of −3.5D (−7.37D, +3.5D) had significantly more myopia cases (*p* = 0.007) than SV spectacle lenses with a median of −1.25D (−9.75D, 0.88D). PALs (median:−3.25D (−6.625D, −0.75D)), MC spectacle lenses (median: −2.25D (−7.38D, 0.25D)), ortho-K lenses (median: −2.5D (−6.75, 0.375)), and unclassified (median: −1.88D (−8.63D, 0.13D)) showed significantly more myopia cases than SV spectacle lenses and MWC groups (*p* < 0.001). The SER distributions among MC products (MC spectacle lenses, MCCL, Ortho-K lens, atropine, and PALs) were similar (*p* = 0.99).

**Figure 4:**
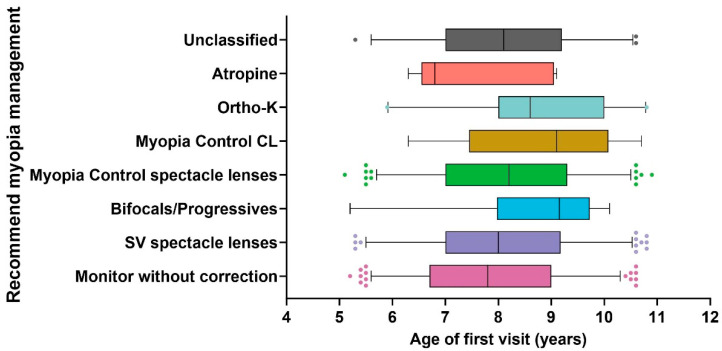
Age distribution in recommended myopia management. Box–whisker plot with 2.5% to 97.5% percentile. The scattered points are referred to as Outliers.

Children with PALs were older than those with MWC (*p* = 0.016). The median age of children with MWC was 7.8 (5.2, 10.6) years (*p* < 0.001) and that of children with SV spectacle lenses was 8 (5.3, 11.5) years (*p* = 0.018), and they were younger than the Ortho-K lens group, with a median of 9.1 (6.3, 10.7) years old. The rest of the groups had a similar age distribution (Figure 4).

### 4.3. The First Modification of Myopia Management

Over 5 years, 104 myopes and 97 pre-myopes were recommended to modify their myopia management. Among them, 82.7% of children in the myopia group and 54.6% of children in the pre-myopia group were advised to control their myopia.

Age of the first visit had a significant correlation with SER change from the first visit to the next recommendation update in the pre-myopia group (r = 0.27, *p* = 0.013) but not in the myopia group (*p* > 0.05). Younger age at the first visit was associated with less myopia progression of pre-myopes when their myopia management was suggested to be modified.

In the myopia group, children who were recommended using Ortho-K lenses (a median of 0.7 years (0.1, 3.1 years)) had shorter recommendation updates than children recommonded changing to use MC spectacle lenses (median of 1.1 years (0.3, 3.3 years)) (after the Bonferroni correction and Kruskal–Wallis test, *p* = 0.01). However, there was no statistically significant difference in time for the next recommendation update among the myopia management after the Bonferroni correction (*p* > 0.05) in the pre-myopia.

In the pre-myopia group, the majority of the updated recommendations were SV spectacles (43.9%) and myopia control spectacles (37.9%). In the myopia group, the majority of updated recommendations were SV spectacles (24.1%), myopia control spectacles (53.9%), and Ortho-K lenses (11.7%). No statistically significant difference in SER changes among the different myopia management was observed in both myopia and pre-myopia groups (*p* > 0.05).

## 5. Discussion

This study is the first to examine the pattern of myopia management in Hong Kong. We investigated 10 clinics across the three parts of Hong Kong: Hong Kong Island, Kowloon, and New Territories. The optometrists who took part in the study have a broad spectrum of experience, with their graduation years spanning from 1983 to 2016, and these clinics have established myopia management for over 10 years.

Practitioners from 10 clinics participated in the survey and cases of 1326 children from 2017 to 2021 were reviewed. In 2017, only MyoKids and MyoVision from ZEISS was recommended as myopia control spectacle lenses. Notably, myopia control spectacle lenses, such as DIMS (with the brand name MiyoSmart) spectacle lenses, were first introduced in Hong Kong in August 2018 [16]. The DIMS spectacle lens is a dual-focus spectacle lens consisting of a central optical zone for distance correction and incorporates a series of small segments of + 3.50D for inducing clear vision and myopic defocus simultaneously for myopia control [16]. It was a totally new concept for myopia control. The recommendation of myopia control methods has increased since then.

At the first visit from 2017 to 2021, 58.5% of children were prescribed SV spectacle lenses or managed with minimal intervention, and only 37.9% were recommended to control myopia. SV spectacle lenses remained the most popular method on the first visit, which is consistent with a recent survey by the International Myopia Institute (IMI) [10,17]. Over the five-year period, myopia management recommendations shifted significantly. In 2017, only 14.8% of children were advised to pursue myopia control, but this increased threefold to 42.1% by 2021.

Dramatically, the recommendation of MC spectacle lenses accounted for 7.3% in 2017 and increased to the highest proportion with 36.8% in 2021, marking a fivefold rise over the five-year period. In contrast, there was minimal change in the recommendation rate for ortho-K lenses and MCCL during this time. Notably, the recommendation of atropine remained consistently low, accounting for less than 1% (Figure 3).

The practice patterns for myopes and pre-myopes were analyzed separately. For myopes, we found the recommendation of SV spectacle lenses accounted for the highest proportion in 2017, with 44.9%, while it dropped by half to only 22.4% in 2021. Such a finding is similar to a previous study; in a survey in 2015, Wolffsohn and colleagues found Asian practitioners had the highest frequency of prescribing SV spectacle lenses (57.6 ± 31.3%), while Australian practitioners showed the lowest frequency (36.8 ± 30.2%; *p* < 0.001) [10]. In a survey in 2019, although the frequency of prescribing SV spectacle lenses declined to 32.3 ± 29.3% in Asia and 30.1 ± 28.1% in Europe, SV spectacle lenses were still the most frequent myopia management strategy used in most of the continents [17]. The PALs, which were once considered strongly for myopia control methods, accounted for more than 6% and dropped suddenly to less than 1% in 2021. Such changes concurred with newer research evidence suggesting that bifocals and PALs were not effective in slowing down myopia progression [18,19]. On the contrary, the recommendation for myopia control methods (MC spectacle lenses, MCCL, ortho-K lenses, and atropine) increased 3 times from around 20% to 60% over 5 years. In 2017, only 11.4% of children were recommended MC spectacle lenses, while the proportion increased 5 times to 54% in 2021, meaning it has become the most popular management strategy for myopes. The MCCL and ortho-K lenses were not as popular as MC spectacle lenses, with less than 6% recommendation over 5 years. Such findings were similar to the survey conducted in 2018 and 2022 [10,17]. Wolffsohn et al. found a greater frequency of prescribing myopia control spectacles than MCCL to young myopes (overall, 14.0% and 9.0%, respectively). Australasian eye care practitioners prescribed more myopia control CLs to around 40% of young myopes, but this ratio was lower elsewhere, with 30% in Europe and North America, 15% in Asia, and only 10% in South America [10]. We suggest that the low frequency of contact lenses for young children may be primarily due to a misconception regarding safety concerns that young children may have concerning higher risks of corneal infection with poorer hygiene practices. However, it has been demonstrated that children and adolescents are as safe as adults in contact lens wear [20,21,22,23]. In a previous study that reviewed the cases of 3500 CL wearers, Wagner and colleagues found only a 0.1% frequency of serious complication (microbial keratitis) occurrence in both children and teens. Referring to the significant complications, such as infiltrative or mechanical ones, teenage wearers showed higher incidence than children (2.3% vs. 0.9%) [24]. 

The recommendation of atropine was less than 1% consistently. It has been reported that the prescription of the pharmaceutical intervention varied among continents, with the highest percentage in Europe (47%) and the lowest in Asia (7%) [17]. The reason might be that registered optometrists in Hong Kong are not licensed to prescribe atropine; only ophthalmologists in Hong Kong have the authority to prescribe it. The need to refer from optometrists to ophthalmologists may introduce an additional hurdle for myopia management and lead optometrists to recommend other modalities for myopia control. The poor access to low-dose atropine preparations has also been reported in other countries [12].

Regarding the pre-myope children, the majority were recommended MWC, and none were recommended to wear MC spectacle lenses to prevent myopia onset. Some studies have shown lower hyperopia at a young age is a risk factor for myopia development [25]. There is limited evidence on whether implementing myopia control during the pre-myopia stage could prevent myopia onset. This highlights a gap in understanding the potential benefits of early intervention in pre-myopes.

We further investigated the factors that led the optometrist to select different myopia control methods. Children recommended MC approaches had significantly more myopia than children recommended SV spectacle lenses or MWC (Figure 3, *p* < 0.05). With a median of 0.125D, children who were recommended MWC had more hyperopia among all management plans (*p* < 0.001), as children who were prescribed such a method were usually pre-myopia children. Those who were prescribed MCCLs (*p* = 0.007), PALs (*p* < 0.001), MC spectacle lenses (*p* < 0.001), and ortho-K lenses (*p* < 0.001) had more myopia than the SV spectacle lenses group. It seems that more myopia alerted optometrists to use other devices besides SV to retard myopia progression. The SER distributions among MC products (MC spectacle lenses, MC CLs, ortho-K lens, and atropine) were similar (*p* = 1.00), which may indicate that optometrists do not have prioritize one method over the other methods but rather depend on the needs and lifestyle of the child patient.

Children who were recommended SV spectacle lenses (median 8 years old) were younger than those fitted with ortho-K lens (median 9.1 years old) (*p =* 0.018, respectively). The reason might be that people tend to believe that older children can handle contact lenses better than younger children. A similar age range has been reported in a previous study, with an average of 8.6 years for soft contact lenses approved for myopia control and 9.2 years for ortho-K lenses [17].

A younger age at the first visit was associated with less myopia progression in pre-myopes when optometrists recommended adjusting their myopia management. Most of the pre-myopes were suggested to be monitored without correction, while SV spectacles and myopia control spectacles were the top two methods when the pre-myopes started to manage myopia. This suggests that earlier intervention and treatment modifications may be necessary for younger patients. While direct evidence on this is scarce, studies have shown that the earlier onset of myopia increases the risk of developing severe or pathological myopia later in life [26]. Therefore, practitioners should remain vigilant with young pre-myopes and modify their management plans promptly, even if their myopia appears to progress more slowly.

During the case review, we found many practitioners lack the axial length measurement record. Therefore, more professional guidelines and education from the local optometry associations should be conducted to standardize the medical records and essential instrumentation, such as including important parameters like myopia control history, cycloplegic refractive error and axial length measurements. Even within the optometric communities, the awareness of myopia control treatments is not always widespread or prioritized. And some of them have not kept up to date with the latest myopia management skills and knowledge. However, a limitation of our study is that we did not include ophthalmologists; in the future, it would be beneficial to study the practice patterns of ophthalmologists in Hong Kong as well.

The current myopia management methods are more expensive than traditional SV spectacle lenses. In Hong Kong, where myopia is highly prevalent, the government welfare system provides very limited financial support for children. Therefore, it is imperative that health insurance and additional financial support from the government are provided. In addition, raising public awareness, improving access to affordable treatments, and encouraging early intervention are essential to overcoming myopia problems and reducing the growing global myopia burden.

## 6. Conclusions

From 2017 to 2021, myopia management patterns in Hong Kong clinical practices shifted significantly, with an increasing number of children being recommended for myopia control. Myopia control spectacle lenses emerged as the most commonly recommended method, while atropine was the least preferred option. Practitioners tended to modify myopia management strategies earlier for pre-myopes who were of a younger age during their first visit.

## Figures and Tables

**Table 1:** Comparisons of myopes (*n* = 859) and pre-myopes (*n* = 459) based on their first visit during 2017–2021.

Parameters	Myopes	Pre-Myopes	*p* Value ※
Age (year)	8.2 ± 1.4	7.8 ± 1.5	<0.001 ^#,^*
AL (mm)	24.15 ± 0.98	23.04 ± 0.78	<0.001 ^#,^*
BCVA	21 ± 2	21 ± 2	0.688 ^#^
Sex (Male/Female/Unknown)	416/417/26	223/216/20	0.814 º
SER (D)	−2.25 ± 1.45	−0.16 ± 1.37	<0.001 ^#,^*
J_0_ (D)	0.36 ± 0.44	0.17 ± 0.24	<0.001 ^#,^*
J_45_ (D)	−0.03 ± 0.15	−0.02 ± 0.09	0.037 ^#,^*

AL: axial length; BCVA: best-corrected visual acuity; SER: spherical equivalent refractive error; D: diopter; J_0_: cylinder power set at orthogonally 90° to 180° meridians, representing Cartesian astigmatism; J_45_: a cross-cylinder set at 45° to 135°, representing oblique astigmatism; ※: comparison between myopia and pre-myopia group; ^#^: Mann–Whitney test; º: Pearson’s chi-squared test; *: *p* < 0.05, reaching the significance level.

**Table 2:** Brand name of the lens was recommended.

Myopia Recommendation	Brand Name of the Lens
Myopia control spectacle lenses	MyoKids, MyoVision (ZEISS), MiyoSmart (HOYA), Stellest (Essilor)
Bifocal/progressive spectacle lenses	Myopliux (Max, Plus), KidsPro (SWISSCOAT)
Myopia control contact lenses	MiSight (CooperVision)

## Data Availability

The datasets and codes used within this paper are available from the corresponding author upon reasonable request.

## Supplementary Materials

The following supporting information can be downloaded at: https://www.mdpi.com/article/10.3390/jcm14030698/s1, Figure S1: The chart for selecting and filling in subject ID. For each calendar year between 2017 and 2021, 5 files were reviewed for each age between 6 and 10, thus there were 125 patients (See Figure 1). Two groups (myopes and pre-myopes) of patients were investigated. Therefore, around 250 patients were reviewed from each optometry practice. For each unique (non-shaded cell) there were tally of 5 unique charts for review. For example, 5 charts of children born in 2011 with refractive error ≤ +0.75D, seen in 2017, 5 charts of children born in 2010 with refractive error ≤ +0.75D, seen in 2017. Likewise, the selection for myopes group were the same. The demographic data, refractive error of children as well as the myopia management recommended by the optometrists were reviewed; Figure S2: Frequency of graduation year of participated optometrists; Figure S3: Line diagram of changes in recommended myopia management from 2017 to 2021.
