# Supplementary material for: Myopia Management in Hong Kong
Source: J Clin Med. 2025 Jan 22;14(3):698. doi: 10.3390/jcm14030698 (PMC11818885; doi:10.3390/jcm14030698)
Supplement: Supplementary file 1 [file jcm-14-00698-s001.zip › jcm-3339243-supplementary.pdf]

		Year of Clinic Visit									
Birth Year	Age	2017	2018	2019	2020	2021	Part 1	Part 2	Part 3	Part 4	Part 5
2017 Visit											
2011	6	5 unique charts per cell					A01	A02	A03	A04	A05
2010	7						A06	A07	A08	A09	A10
2009	8						A11	A12	A13	A14	A15
2008	9						A16	A17	A18	A19	A20
2007	10						A21	A22	A23	A24	A25
2018 Visit											
2012	6						A26	A27	A28	A29	A30
2011	7						A31	A32	A33	A34	A35
2010	8						A36	A37	A38	A39	A40
2009	9						A41	A42	A43	A44	A45
2008	10						A46	A47	A48	A49	A50
2019 Visit											
2013	6						A51	A52	A53	A54	A55
2012	7						A56	A57	A58	A59	A60
2011	8						A61	A62	A63	A64	A65
2010	9						A66	A67	A68	A69	A70
2009	10						A71	A72	A73	A74	A75
2020 Visit											
2014	6						A76	A77	A78	A79	A80
2013	7						A81	A82	A83	A84	A85
2012	8						A86	A87	A88	A89	A90
2011	9						A91	A92	A93	A94	A95
2010	10						A96	A97	A98	A99	A100
2021 Visit											
2015	6						A101	A102	A103	A104	A105
2014	7						A106	A107	A108	A109	A110
2013	8						A111	A112	A113	A114	A115
2012	9						A116	A117	A118	A119	A120
2011	10						A121	A122	A123	A124	A125

Figure S1. The chart for selecting and filling in subject ID. For each calendar year between 2017 and 2021, 5 files were reviewed for each age between 6 and 10, thus there were 125 patients (See Figure 1). Two groups (myopes and pre-myopes) of patients were investigated. Therefore, around 250 patients were reviewed from each optometry practice. For each unique (non-shaded cell) there were tally of 5 unique charts for review. For example, 5 charts of children born in 2011 with refractive error ≤ +0.75D, seen in 2017, 5 charts of children born in 2010 with refractive error ≤ +0.75D, seen in 2017. Likewise, the selection for myopes group were the same. The demographic data, refractive error of children as well as the myopia management recommended by the optometrists were reviewed.

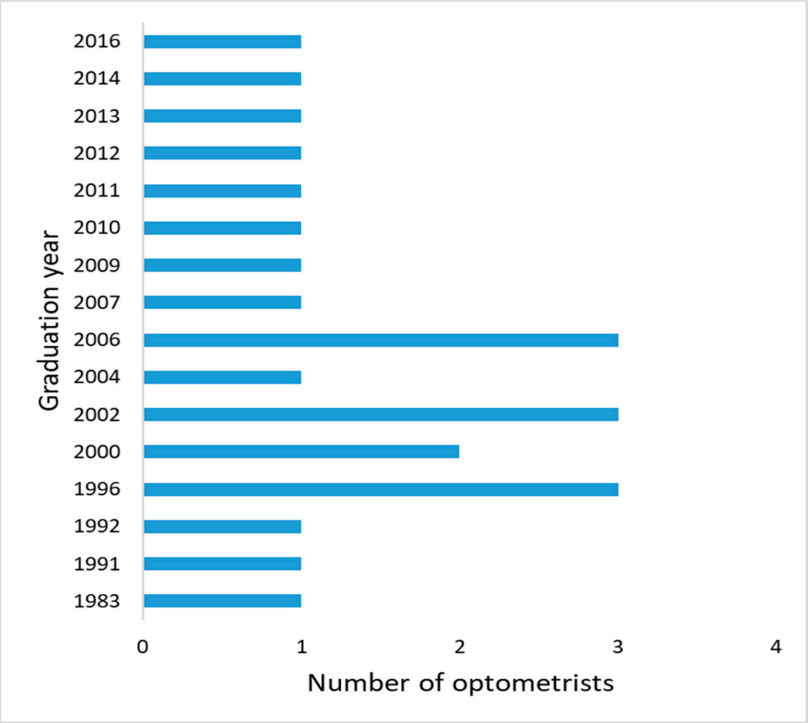

Figure S2. Frequency of graduation year of participated optometrists.

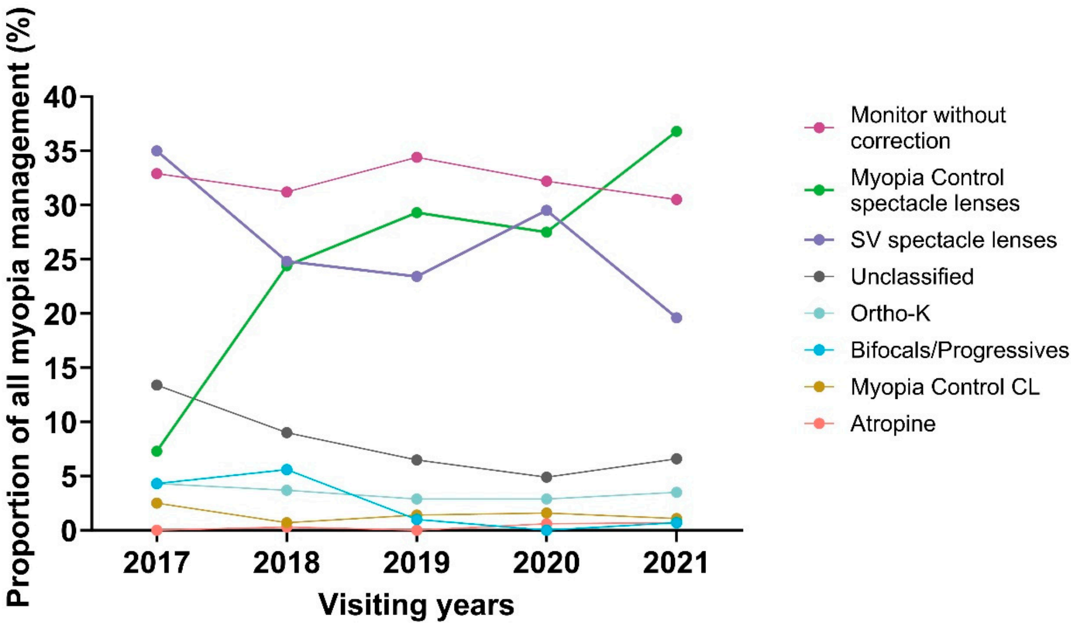

Figure S3. Line diagram of changes in recommended myopia management from 2017 to 2021.
